# Developments in Neurodegenerative Disorders: Highly Cited Articles Published in Brain Sciences in 2023–2024

**DOI:** 10.3390/brainsci15040345

**Published:** 2025-03-27

**Authors:** Mamede de Carvalho

**Affiliations:** 1 Medicine ULisboa for Health Clinical Research and Innovation (MUHCRI), Centro de Estudos Egas Moniz, Faculdade de Medicina, Universidade de Lisboa, 1649-028 Lisbon, Portugal; mamedemg@mail.telepac.pt; Tel./Fax: +351-21-7805219; 2 Department of Neurosciences and Mental Health, ULS de Santa Maria, 1649-028 Lisbon, Portugal

## 1. Introduction

Neurodegenerative disorders, including Alzheimer’s disease (AD), Parkinson’s disease (PD), and amyotrophic lateral sclerosis (ALS), pose a significant and growing health concern, particularly in developed countries. AD, the most common cause of dementia, currently affects an estimated 7 million Americans aged 65 and older [1]. While AD’s precise causes and underlying mechanisms remain incompletely understood, age and genetics are considered the most prominent risk factors. However, cardiovascular health (including conditions like high blood pressure, diabetes, and smoking), head injury, depression, and sleep disturbances also contribute significantly to the risk of AD. Pathologically, AD is characterized by the accumulation of beta-amyloid plaques, tau neurofibrillary tangles, and neuronal loss [2]. The recent modest clinical results observed with monoclonal antibody treatments targeting brain amyloid raise concerns about the central role of beta-amyloid plaques in AD onset and progression, indicating that new mechanisms should be investigated [3]. Sleep disturbances, potentially linked to neurotoxicity from glymphatic dysfunction [4,5], are associated with both AD and PD. PD, a multisystem disorder, currently lacks disease-modifying therapies, highlighting the urgent need for new treatment strategies. Vascular risk factors can contribute to parkinsonism and negatively impact PD prognosis, often accompanied by cognitive decline [6]. The complex interplay between vascular impairment, neuroinflammation, and neurodegeneration [7] underscores the need for novel therapeutic approaches; this holds particular significance in ALS, a disease for which, despite numerous clinical trials, no drug has yet proven broadly effective [8].

## 2. The Role of Tryptophan Metabolism in Alzheimer’s Disease

Savonije and Weaver highlight the potential of tryptophan in AD treatment. Tryptophan, a crucial amino acid, is metabolized into serotonin, a neurotransmitter influencing mood, personality, and pain perception—all common targets of SSRIs. However, tryptophan also follows the Kynurenic Pathway, impacting brain immune responses and neuroinflammation. Intriguingly, both pathways influence neprilysin activity, an enzyme connected to amyloid-beta expression. Furthermore, tryptophan supplementation can induce sedation, and its metabolites regulate sleep, potentially offering cognitive protection. Therefore, as this contribution suggests, exploring small molecules that modulate these pathways holds significant promise for AD therapy.

## 3. Hypothalamus and Post-Traumatic Stress Disorder: A Review

Raise-Abdullahi et al. review the critical link between the hypothalamus and post-traumatic stress disorder (PTSD). The hypothalamus, a small but central brain structure, comprises diverse neuronal clusters with wide-ranging functions, including control of the autonomic nervous system, sleep, temperature regulation, appetite, thirst, sexual drive, emotions, pain responses, memory, and endocrine activity. PTSD, a prevalent psychiatric disorder affecting a significant portion of the modern population, poses a serious challenge, particularly for vulnerable individuals. This disorder can disrupt hypothalamic function, impacting control over the autonomic nervous system, sleep, and neuroendocrine axes. The authors effectively detail the endocrine dysfunctions associated with PTSD, citing key studies in this area. While the review focuses on PTSD, it is important to note that hypothalamic dysfunction has also been implicated in other neurodegenerative diseases like AD [9], PD [10], and ALS [11], further highlighting the significance of this review.

## 4. Could Vitamins Have a Positive Impact on the Treatment of Parkinson’s Disease?

PD is the second most common neurodegenerative disorder, characterized by the intracellular accumulation of alpha-synuclein protein in Lewy bodies and the loss of dopaminergic neurons in the substantia nigra pars compacta. Its etiology is linked to genetic mutations, environmental toxins, and oxidative stress. While a prior study investigated vitamin E supplementation as an antioxidant treatment to slow disease progression, it yielded negative results [12].

Sahu et al. reviewed the potential protective role of vitamins in PD. While retinol (vitamin A) possesses anti-inflammatory and anti-apoptotic properties that could theoretically protect dopaminergic neurons, clinical evidence of its efficacy is lacking. Thiamine (vitamin B1) shows greater promise, as chronic low intake may be a risk factor for parkinsonism [13]; however, evidence of supplementation benefits remains limited. Pyridoxal 5′-phosphate deficiency is implicated in several neurological disorders, including PD and AD, making vitamin B6 a subject of considerable interest. Studies suggest an increased risk of PD with low vitamin B6 intake and a decreased risk with high dietary intake [14]. Vitamin B12 deficiency can induce various neurological conditions, including parkinsonism with cognitive impairment. Some evidence indicates that vitamin B12 supplementation may positively influence PD prognosis [15]. Ascorbic acid (vitamin C), with anti-inflammatory and antioxidant properties, has been linked to PD risk, with deficiency increasing risk and supplementation potentially decreasing it and improving prognosis. Vitamin D levels often decrease in PD patients, a common finding in the elderly; further research is required to determine the clinical impact of vitamin D3 supplementation. Vitamin E has been extensively studied, and high intake reduces PD risk. However, despite some positive findings, its therapeutic value remains questionable [12]. Vitamin K, particularly vitamin K2, may improve mitochondrial function, which is relevant to PD etiopathogenesis, especially in patients with PINK1 mutations; further research is needed to establish its clinical utility.

## 5. Visual Dysfunction in Parkinson’s Disease

Nieto-Escamez et al. examine the multifaceted area of visual dysfunction in PD, encompassing ocular impairments (including visual acuity, contrast sensitivity, and color vision), oculomotor control deficits (like as blepharospasm), visuoperceptual and visuospatial impairments, and perceptual disturbances, including visual hallucinations. They thoroughly discuss the neuroanatomical correlations, incorporating neuroimaging findings and their relationship to the PD phenotype and genetic profile; this is an important contribution in this era.

## 6. Neuroprotective Potential of Flavonoids in Brain Disorders

Flavonoids, a large subgroup of polyphenols in fruits, vegetables, bark, and herbs, are recognized for their antioxidant properties. Given their ability to cross the blood–brain barrier, flavonoids hold potential therapeutic applications for various neurological conditions. Hasan et al. comprehensively review this topic, detailing flavonoid pharmacology and therapeutic mechanisms, particularly their anti-inflammatory effects. The authors also explore their antiplatelet actions, which reduce the risk of thrombi, and summarize current evidence regarding flavonoid use in stroke prevention and therapy. Notably, the review carefully addresses the potential toxicity of flavonoids, presenting a well-balanced assessment.

## 7. Trimethylamine N-Oxide Exacerbates Neuroinflammation and Motor Dysfunction in an Acute MPTP Mice Model of Parkinson’s Disease

The link between gut microbiome dysbiosis and PD is well-established [16]. Trimethylamine N-oxide (TMAO), a gut microbiota metabolite that can cross the blood–brain barrier, is implicated in PD progression. Quan et al. explored the effects of elevated circulating TMAO on behavioral disorders, dopaminergic neurons, neurotransmitter levels, and neuroinflammation in MPTP-induced PD mice by administering exogenous TMAO. This study addresses the inconsistent findings of elevated plasma TMAO in PD patients, which are often associated with disease severity. The authors demonstrated that TMAO significantly activated microglia and astrocytes in the striatum and hippocampi of PD mice and increased pro-inflammatory cytokine levels in the hippocampi; this led to impaired motor function, although no direct effect on dopaminergic neurons was observed. These findings warrant further investigation into the role of TMAO in PD.

## 8. Reelin Signaling in Neurodevelopmental Disorders and Neurodegenerative Diseases

Reelin, an extracellular matrix glycoprotein, is crucial in neuronal migration during embryonic brain development. Dysfunction at this stage is associated with neurodevelopmental disorders, including cognitive impairment, autism, ataxia, and psychiatric disorders such as schizophrenia. In the adult brain, Reelin is essential for synaptic plasticity, particularly the potentiation of glutamatergic and GABAergic neurotransmission. Given its significant influence on synaptic function and brain plasticity, its role in memory and learning disorders is a rapidly expanding area of research. This review by Joly-Amado summarizes the literature on Reelin’s involvement in physiological aging and neurodegenerative disorders, exploring its potential as a therapeutic target for preventing or treating neurodegeneration.

## 9. The Interplay Between Mitochondrial Dysfunction and Ferroptosis During Ischemia-Associated Central Nervous System Diseases

As outlined in the introduction, cerebral ischemia, beyond its role in stroke, is a risk factor for AD and is associated with parkinsonism. Ischemic brain tissue damage disrupts mitochondrial function, including the respiratory chain and membrane integrity, releasing pro-apoptotic factors. This damage also initiates neuronal lesions through autophagy and ferroptosis. Ferroptosis, a regulated cell death mechanism, is characterized by iron-dependent accumulation of reactive oxygen species and lipid peroxidation. Iron accumulation within mitochondria during ischemia exacerbates oxidative stress, potentially accelerating ferroptosis and impairing neuronal survival. However, the precise relationship between mitochondrial dysfunction and ferroptosis in brain ischemia remains incompletely understood. This review by Tian et al. addresses this complex interplay, specifically focusing on AD, Parkinson’s disease (PD), stroke, and epilepsy.

## 10. Exploring Whether Iron Sequestration Within the CNS of Patients with Alzheimer’s Disease Causes a Functional Iron Deficiency That Advances Neurodegeneration

In an original experimental study, LeVine et al. investigated the involvement of iron in the pathogenesis of AD. They examined the frontal cortex of postmortem brain tissue from eight individuals with AD and six individuals who died from other causes. The study revealed a high degree of iron binding to plaques and tangles within the cortical sections of AD patients. This increased binding suggests a reduction in iron bioavailability for essential biochemical processes. Further supporting this hypothesis, the authors demonstrated elevated expression of protein-coding transcripts associated with anemia-like responses. The researchers posit that this induced iron deficiency state may contribute to AD pathogenesis by promoting neuronal death. Validation of these preliminary findings could potentially unlock new therapeutic avenues for AD.

These highly cited articles in Brain Sciences address key topics in neurodegeneration. Neurodegenerative diseases pose a significant and increasing global health challenge while also serving as crucial models for scientific discovery aimed at improving quality of life. As advocates for open science, we encourage readers to explore these valuable contributions and contribute to further research in this field.
